# Histological and biochemical changes in lymphatic vessels after skeletal muscle injury induced by lengthening contraction in male mice

**DOI:** 10.14814/phy2.15950

**Published:** 2024-02-14

**Authors:** Yuma Tamura, Takafumi Kawashima, Rui‐Cheng Ji, Nobuhide Agata, Yuta Itoh, Keisuke Kawakami

**Affiliations:** ^1^ Physical Therapy Research Field, Graduate School of Medicine Oita University Yufu Japan; ^2^ Department of Rehabilitation Akeno‐Central Hospital Oita Japan; ^3^ Faculty of Welfare and Health Science Oita University Oita Japan; ^4^ Faculty of Health and Medical Sciences Tokoha University Hamamatsu Japan; ^5^ Faculty of Rehabilitation Science Nagoya Gakuin University Nagoya Japan

**Keywords:** inflammation, lengthening contraction, lymphatic vessels, skeletal muscle injury

## Abstract

Lymphatic vessels are actively involved in the recovery process of inflamed tissues. However, the changes in intramuscular lymphatic vessels during inflammation caused by skeletal muscle injury remain unclear. Therefore, the purpose of this study was to clarify the changes in lymphatic vessels after skeletal muscle injury. The left tibialis anterior muscles of male mice were subjected to lengthening contractions (LC) for inducing skeletal muscle injury, and samples were collected on Days 2, 4, and 7 for examining changes in both the skeletal muscles and intramuscular lymphatic vessels. With hematoxylin–eosin staining, the inflammatory response was observed in myofibers on Days 2 and 4 after LC, whereas regeneration of myofibers was found on Day 7 after LC. The number and area of intramuscular lymphatic vessels analyzed by immunohistochemical staining with an antibody against lymphatic vessel endothelial hyaluronan receptor 1 were significantly increased only on Day 4 after LC. Based on the abovementioned results, intramuscular lymphatic vessels undergo morphological changes such as increase under the state of muscle inflammation. This study demonstrated that the morphology of intramuscular lymphatic vessels undergoes significant changes during the initial recovery phase following skeletal muscle injury.

## 1 INTRODUCTION

Skeletal muscle damage caused by excessive strain in daily life and sports can lead to disability, functional limitations, and reduced quality of life (Greising et al., 2020). Recovery of injured skeletal muscles is a gradual process, and there is growing interest in accelerating the return to normal daily activities and sports performance. Several cells may be involved in recovery from muscle injury, including inflammatory cells such as neutrophils and macrophages, muscle satellite cells from skeletal muscle‐specific stem cells, and fibroadipogenic progenitor cells derived from mesenchymal stromal and endothelial cells (Chongsatientam & Yimlamai, 2016; Deasy et al., 2009; Dort et al., 2019; Dueweke et al., 2017; Hawke & Garry, 2001; Mierzejewski et al., 2020; Molina et al., 2021; Teixeira & Duarte, 2016). Notably, macrophages are closely involved in recovery from skeletal muscle injury; these cells usually accumulate at the injured site and release various cytokines for removing debris and activating skeletal muscle satellite cells (Dort et al., 2019; Dueweke et al., 2017). Importantly, macrophages may influence various pathological processes, from the induction to convergence resolution of an injury‐induced inflammatory response by promoting lymphangiogenesis (Ji, 2012).

Lymphatic vessels collect excess interstitial fluid and play a role in maintaining tissue homeostasis (Oliver et al., 2020). In addition, lymphatic vessels are involved in the recovery process of inflamed tissues. For example, lymphatic vessels were found to be increased and expanded in the inflammatory tissues of dermatitis and arthritis, which suggested to promote inflammatory cell migration and tissue repair (Guo et al., 2009; Huggenberger et al., 2010). However, the changes in the distribution and morphology of intramuscular lymphatic vessels after skeletal muscle injury are unknown, and their role in muscular inflammatory responses remains also unclear.

In the first place, there have been few reports on the localization of lymphatic vessels in skeletal muscle (Kivelä, Havas, & Vihko, 2007; Kivelä, Silvennoinen, et al., 2007), and even the distribution of lymphatic vessels in normal skeletal muscle is unknown.

Naturally, there is no detailed examination of the pathophysiology of lymphatic vessels in skeletal muscles. In a recent study, we (Kawashima, Ji, et al., 2021) reported that lymphatic vessels in soleus muscle are adjacent to blood capillaries and widely distributed between myofibers. We also reported that 4 weeks of tail suspension reduced the number of lymphatic vessels in the soleus muscle. Furthermore, Pu et al. (2021) reported that intramuscular lymphatic vessels are actively involved in the recovery of ischemic skeletal muscle.

Recent studies have shown that lymphatic vessels are increased by lymphatic endothelial growth factors, vascular endothelial growth factor (VEGF)‐C, VEGF‐D, and their receptors (VEGFR)‐3 (Guo et al., 2009; Huggenberger et al., 2010; Ji, 2012; Skobe et al., 2001; Stacker et al., 2001; Zhang et al., 2007). These growth factors are also induced by inflammatory cytokines that are released from macrophages, such as tumor necrosis factor (TNF)‐α and interleukin (IL)‐1β (Ji, 2012; Zhang et al., 2007). We expect that the inflammation that occurs during skeletal muscle injury may contribute to lymphangiogenesis.

It seems obvious that lymphatic vessels may change their distribution and morphology during skeletal muscle injury, and thus play an important role in the regulation of skeletal muscle pathology. In this way, they may also have a significant effect on recovery from skeletal muscle injury and inflammatory response. The purpose of this study was to examine histological and biochemical changes in lymphatic vessels after skeletal muscle injury. In addition, clarify the relationship between the histological characteristics of intramuscular lymphatic vessels and skeletal muscle injury recovery for providing a new insight into the therapeutic development in the field.

## 2 MATERIALS AND METHODS

### 2.1 Animals

All experiments were approved by the animal ethics committee of Oita University (No. 185701). Ten‐week‐old C57BL/6 mice (Japan SLC, Shizuoka, Japan) were used for this study. Furthermore, it is expected that results will vary by sex, including hormonal effects (Serra et al., 2013; You et al., 2023). Therefore, our experiments were specifically conducted using male mice. All mice were housed at room temperature (RT, 25°C) under a 12‐h light–dark cycle and were provided with food and water ad libitum.

To induce skeletal muscle injury, lengthening contractions (LC) were conducted on the left tibialis anterior (TA) muscle of mice. To assess the degree of myofibers damaged by LC histologically, hematoxylin and eosin (H–E) staining and Evans blue dye (EBD) staining were performed. First, 1% EBD (Fujifilm Wako Pure Chemical Co., Osaka, Japan) in phosphate‐buffered saline (PBS) was administered intraperitoneally at a dose of 1% of body weight 24 h after LC (Hamer et al., 2002; Mori et al., 2014), and then the TA muscles were collected 24 h later (LC_EBD, *n* = 10). We also created a group that only received intraperitoneal injection of EBD, but without LC injury (CON_EBD, *n* = 8).

To assess the histological and biochemical changes in myofibers, lymphatic vessels, and blood capillaries after LC, we divided mice into four groups: to collect TA muscle 2 days after LC (2 day, *n* = 13), 4 days after LC (4 day, *n* = 13), 7 days after LC (7 day, *n* = 13), and non‐LC group (CON, *n* = 12).

At the end of the experimental period, the mice were euthanized by cervical dislocation. For histological analysis, the TA muscles were collected and immediately frozen in isopentane precooled in liquid nitrogen, whereas, for biochemical analysis, the same muscles were directly frozen in liquid nitrogen. These samples were stored at −80°C until further analysis.

### 2.2 Lengthening contractions

**FIGURE 1:**
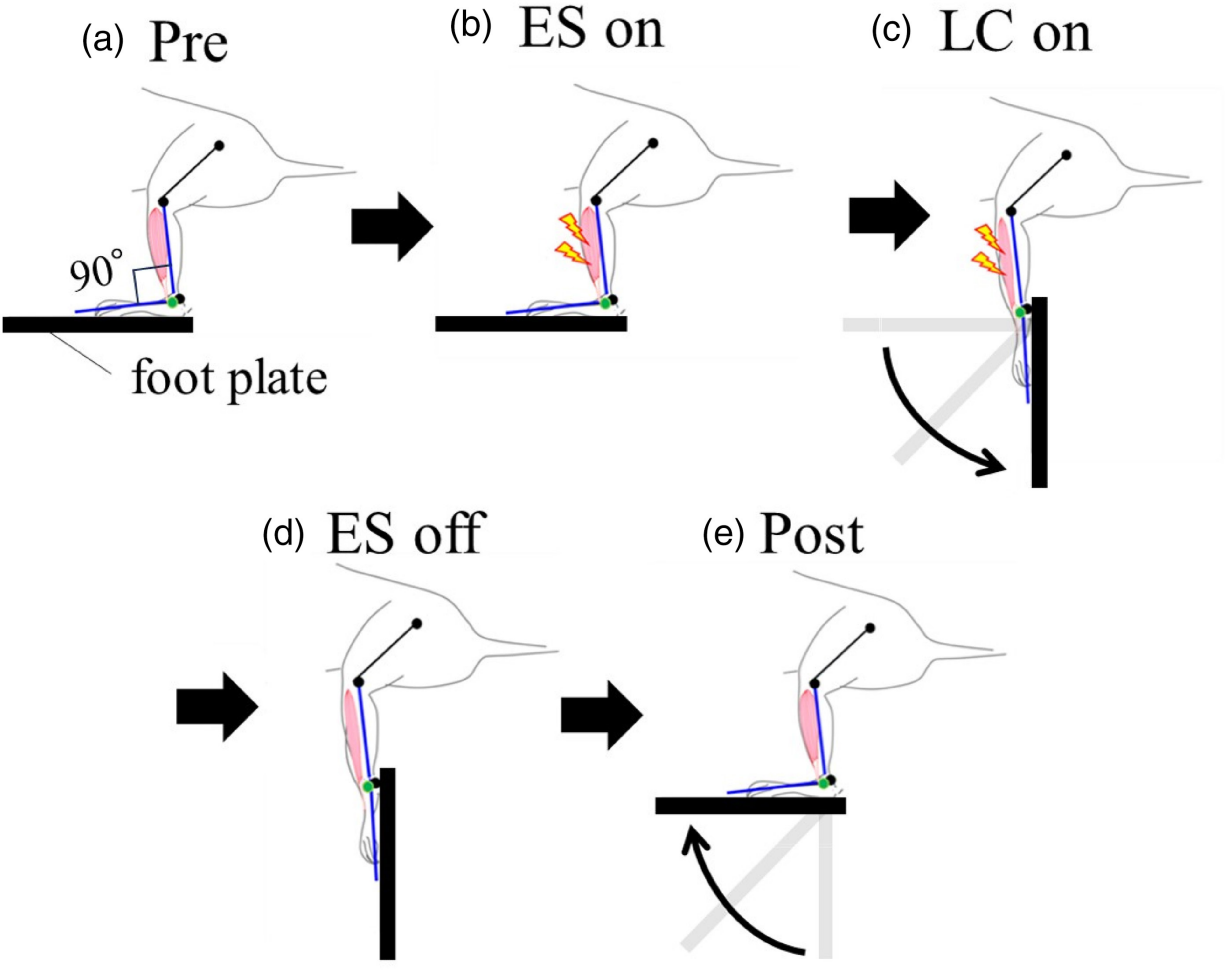
LC methods. Electrical stimulation (ES) is applied to the left TA muscle of a mouse secured to the device (a) to induce maximal isometric contraction of the ankle dorsiflexor muscle group (b). Two hundred milliseconds after initiating EC, the footplate rotates 90° at an angular velocity of 800°/s, initiating LC against the TA muscle (c). After 113 ms of LC, EC is terminated (d), and the limb returns to its initial position (e).

Considering that the mechanism of muscle damage in clinical practice is often caused by eccentric contraction, which occurs when a muscle lengthens as it contracts (Teixeira & Duarte, 2016), we employed an LC‐induced skeletal muscle injury model, which holds greater clinical relevance in this study. Therefore, we conducted LC on mouse TA muscle, modifying a previously reported method (Mori et al., 2014, 2018). Specifically, mice under inhalation anesthesia with isoflurane (1.4%; Fujifilm Wako Pure Chemical Co.) were fixed to a customized device (ODF‐1; Bio Research Center, Nagoya, Japan), and the sole of the mouse was attached to the foot plate via adhesive tape. In addition, the angle of the ankle joint was set so that the line connecting the footplate and fibula formed a right angle (Figure 1a). To provide the electrical stimulation necessary for skeletal muscle contraction, surface electrodes (Bio Research Center) with a diameter of 3 mm were attached with adhesive gel at two locations (The first location: 1 mm anterior and 1 mm distal to the fibular head. The second location: 3 mm distal to the first location.). An electric current (1.5 mA, 100 Hz) was applied transcutaneously to the dorsiflexors of the ankle joint using an electric stimulator (SEN‐3301; Nihon Kohden, Tokyo, Japan), and the dorsiflexors of the ankle joint were contracted isometrically (Figure 1b). Two hundred milliseconds after the start of electrical stimulation, the foot plate was rotated in the plantar flexion direction with a range of motion of 90° and an angular velocity of 800°/s (Figure 1c). After the stimulation was stopped and held for 100 ms (Figure 1d), the foot plate was returned to the starting position at an angular velocity of 200°/s (Figure 1e). In this condition, 150 times LCs were conducted with a pause time of 1 s.

### 2.3 Functional test

To evaluate the function of skeletal muscle, we measured the maximum torque when the ankle dorsiflexor muscle group was contracted isometrically tetanic (Mori et al., 2014, 2018). The device used to measure the maximum torque, the position of electrode attachment, and the electrical stimulation conditions were conducted in the same way as the LC method. The protocol for positioning the mouse on the device also adhered to that of the LC method. The maximum torque was measured byss applying transcutaneous electrical stimulation to the ankle dorsiflexor muscle group and contracting it isometrically to a maximum. The maximum torque was measured before LC, 2 days, and immediately before TA muscle collection.

### 2.4 Histological and immunohistochemical analysis

Six samples per group were used for histological and immunohistochemical analyses. Frozen transverse sections of 8 μm thickness were obtained from at 3 mm proximal to the TA muscle at −25°C using a cryostat (CM1860; Leica Biosystems, Nussloch, Germany). The sections were fixed in 4% paraformaldehyde (PFA) (Fujifilm Wako Pure Chemical Co.) in PBS for 10 min, washed in PBS, and stained with H–E. The stained sections were sealed in 90% glycerol and observed under a light microscope (IX70; Olympus, Tokyo, Japan). A 10× objective lens (UPLFLN; Olympus, Tokyo, Japan) was used to obtain images. The observed images were photographed with an attached digital camera (DsRi1; Nikon, Tokyo, Japan) and saved as digital data. Dedicated imaging software was used to capture images (NIS Elements; Nikon, Tokyo, Japan).

Sections from the LC_EBD and CON_EBD groups were conducted to immunohistochemical staining for laminin to identify myofibers. The sections were fixed with PFA for 10 min, washed with PBS, and blocked with 4% Block Ace powder (KAC, Kyoto, Japan) for 60 min at RT. These sections were incubated overnight at 4°C with the primary antibodies, rabbit anti‐laminin antibody (1:40, LSL‐LB‐1013, Lot No. 832011; Cosmo Bio, Tokyo, Japan; RRID:AB_1962701). Sections were washed in PBS and further incubated in the dark for 60 min at RT with Alexa Fluor 488‐conjugated goat anti‐rabbit immunoglobulin G (IgG) (1:400, A‐11008, Lot No. 1829924; Thermo Fisher Scientific, Waltham, US‐MA; RRID:AB_143165). The stained sections were sealed in 90% glycerol and observed under a fluorescence microscope (IX70; Olympus). A 4× objective lens (UPLFLN; Olympus) was used to acquire images. The observed images were photographed using an attached digital camera (DsRi1; Nikon) and saved as digital data. Dedicated imaging software was used to capture images (NIS Elements; Nikon). EBD‐positive myofibers were detected by fluorescent microscopy at 568 nm. From these image data, all laminin‐positive and EBD‐positive myofibers were counted using ImageJ software (ver. 1.51) (Rasband, W.S., ImageJ, U.S. National Institutes of Health, Bethesda, Maryland, USA, http://rsb.info.nih.gov/ij/, 1997–2012.) and the number of EBD‐positive myofibers relative to total myofibers (percentage of EBD‐positive myofibers) was calculated.

Sections from the CON group and the 2‐, 4‐, and 7‐day groups were fixed in PFA for 10 min, blocked with 4% Block Ace powder for 60 min at RT, and then immunohistochemically stained to identify lymphatic vessels and capillaries. These sections were incubated overnight at 4°C with the following primary antibodies: rabbit anti‐mouse lymphatic vessel endothelial hyaluronan receptor 1 (LYVE‐1) (1:1000, 103‐PA50AG, Lot No. 1410R23; Relia Tech GmbH, Wolfenbüttel, Germany; RRID:AB_2876870) and rat anti‐mouse CD31 (1:50, 550274, Lot No. 9259767; BD Biosciences, San Jose, US‐CA, CA; RRID:AB_393571) (Kawashima, Ji, et al., 2021). Sections were washed in PBS and further incubated in the dark for 60 min at RT with Alexa Fluor 488‐conjugated goat anti‐rabbit IgG (1:400, A‐11008, Lot No. 1829924; Thermo Fisher Scientific; RRID:AB_143165) and Alexa Fluor 568‐conjugated goat anti‐rat IgG (1:400, A‐11077, Lot No. 2217022; Thermo Fisher Scientific; RRID:AB_2534121). The stained sections were sealed in 90% glycerol and observed under a fluorescence microscope (IX70; Olympus). A 10× objective lens (UPLFLN; Olympus) was used to acquire images. The observed images were photographed with an attached digital camera (DsRi1; Nikon) and saved as digital data. Dedicated imaging software was used to capture images (NIS Elements; Nikon). Based on these image data, the number and area of all lymphatic vessels and blood capillaries were measured using Adobe Photoshop (Adobe, San Jose, US‐CA). Furthermore, the whole muscle cross‐sectional area (mm^2^) of TA muscles was measured using ImageJ, and the number of lymphatic vessels (number of lymph vessels) and capillaries (number of capillaries) within the whole muscle cross‐sectional area was calculated. All assessments of lymphatic vessels and capillaries were conducted by the same individual. We excluded lymphatic vessels located in the perimysium or those distinctly large in size, following the method outlined by Kivelä, Havas, & Vihko (2007).

Immunohistochemical staining of macrophages (*n* = 4) was performed in the CON group as well as 2‐, 4‐, and 7‐day groups. The procedure was similar to that of lymphatic and blood capillary immunohistochemistry. Macrophages were identified using a rat anti‐mouse F4/80 (1:100, ab6640, Lot No. GR3393406‐1; Abcam, Cambridge, UK; RRID:AB_1140040) as the primary antibody. Alexa Fluor 488‐conjugated goat anti‐rat IgG (1:400, A‐11006, Lot No. 2160405; Thermo Fisher Scientific; RRID:AB_2534074) was used as the secondary antibody. In addition, nuclei were stained with 4′,6‐diamidino‐2‐phenylindole (DAPI) using VECTASHIELD PLUS Antifade Mounting Medium with DAPI (Lot No. ZJ0224; Vector Laboratories) as the mounting material. Imaging was conducted using a 20× objective lens (UPLFLN; Olympus) at six random locations (total area of six locations: 1.45656 mm^2^) using a digital camera (DsRi1; Nikon). Based on the obtained digital data, the number of macrophages was determined using ImageJ software (ver. 1.51).

### 2.5 Biochemical analysis

For biochemical analysis, seven and six samples were used in the LC and CON groups, respectively. Real‐Time reverse transcription PCR was used for biochemical analysis using samples collected from CON group and 2‐day, 4‐day, and 7‐day groups. Total RNA was extracted from the TA muscles of mice using Isogen II (Nippon Gene, Tokyo, Japan; Code No, 311‐07361) according to the manufacturer's instructions. RNA concentration was measured using a DS‐11 spectrophotometer (DeNovix, Wilmington, US‐DE). RNA was reverse‐transcribed into complementary DNA using SuperScript VILO Master Mix (Thermo Fisher Scientific; catalog number, 11755050) and real‐time PCR was performed using Fast SYBR Green Master Mix (Thermo Fisher Scientific; catalog number, 4385612) on a Step One Plus RT–PCR machine (Applied Biosystems, Waltham, US‐MA). VEGF‐C, VEGF‐D, VEGFR‐3, TNF‐α, and IL‐1β gene expression levels were analyzed using the relative standard curve method, and the values were normalized to glyceraldehyde‐3‐phosphate dehydrogenase (GAPDH). All primers were purchased from Greiner Bio‐One (Kremsmünster, Austria). The primers used in this study are listed in Supplemental Table S1.

### 2.6 Statistical analysis

Data were expressed as mean ± standard deviation (mean ± SD). Student *t*‐test was performed to compare between the CON_EBD and LC_EBD group. The paired *t*‐test was performed for comparison before LC and after LC. One‐way ANOVA was performed for comparison among CON, 2‐day, 4‐day, and 7‐day groups. The Tukey test was used to analyze the individual means. *P*‐values of <0.05 were considered to indicate statistical significance. All statistical analyses were performed using R Commander (ver. 4.02).

## 3 RESULTS

### 3.1 Skeletal muscle injury model by LC

**FIGURE 2:**
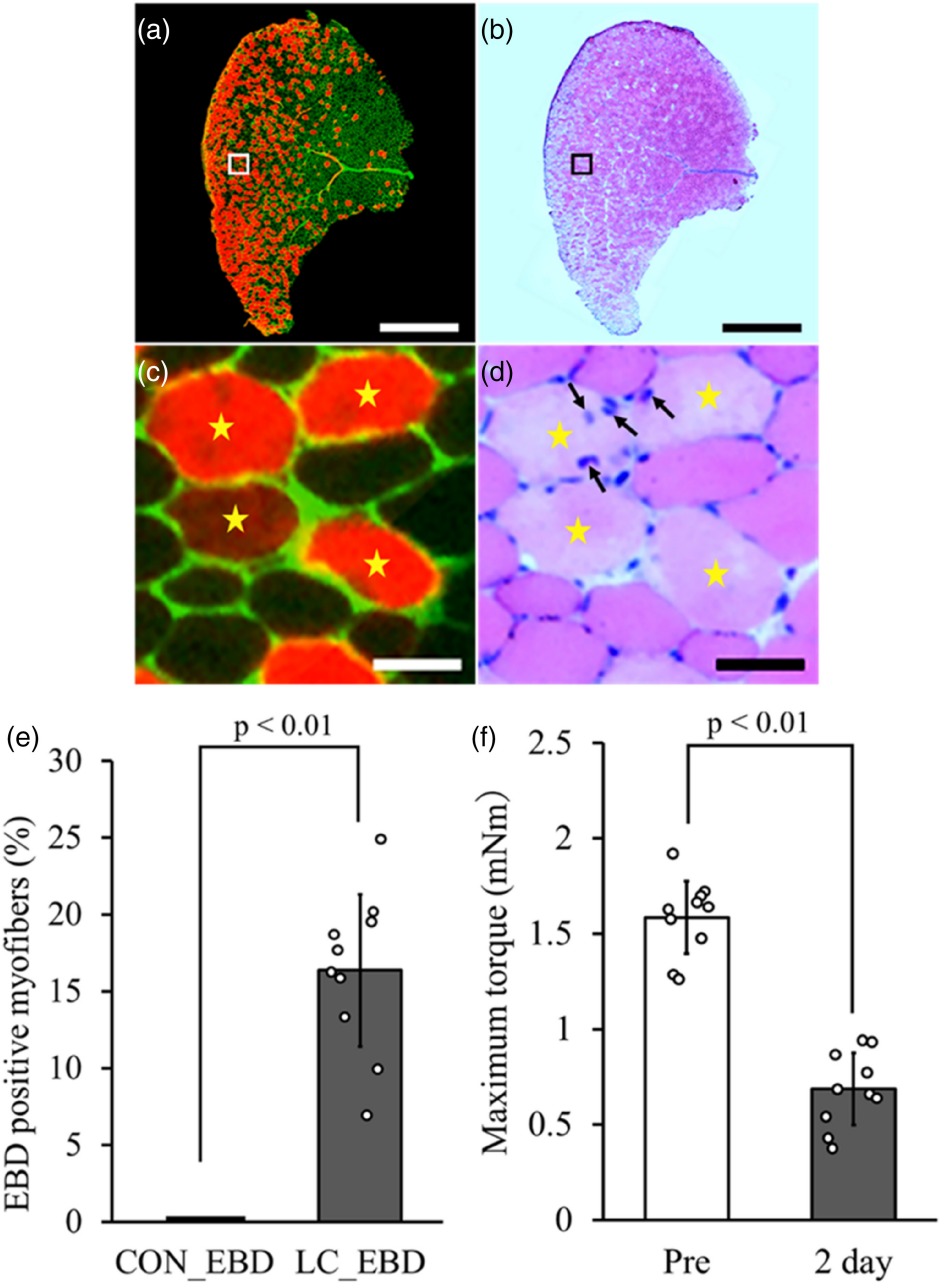
Characteristics of the skeletal muscle injury model. Representative immunohistochemical staining image in the TA muscle of the LC_EBD group (a–d). Immunohistochemical staining (a; green: laminin; red: EBD). H–E staining of sequential sections (b). An enlarged image of the same area (framed section) from images in a and b (c and d). Star markers indicate EBD‐positive myofibers and corresponding myofibers in the H–E‐stained image, whereas arrows indicate nuclei inflow into the myofiber. Scale bars = 1 mm (a and b) and 50 μm (c and d). Percentage of EBD‐positive myofibers in the whole muscle belly (e), and maximum torque before and after LC in the LC_EBD group (f). Data are presented as means ± SD.

In the LC_EBD group, EBD‐positive myofibers in the TA muscle were predominantly distributed in the superficial portion, but fewer in the deep portion (Figure 2a). In addition, in the serial sections, the fluorescent imaging myofibers with EBD uptake showed obvious light color in the H–E staining images in contrast with surrounding myofibers (Figure 2c,d). The percentage of EBD‐positive myofibers was observed at 16.4% ± 4.9% in the LC_EBD group, but hardly observed in the CON_EBD group (Figure 2e). In the LC_EBD group, the maximum torque after LC (0.69 ± 0.19 mNm) was significantly lower than that before LC (1.59 ± 0.19 mNm) by 55.6% ± 14.4% (Figure 2f).

### 3.2 Histological and functional changes in the skeletal muscle

**FIGURE 3:**
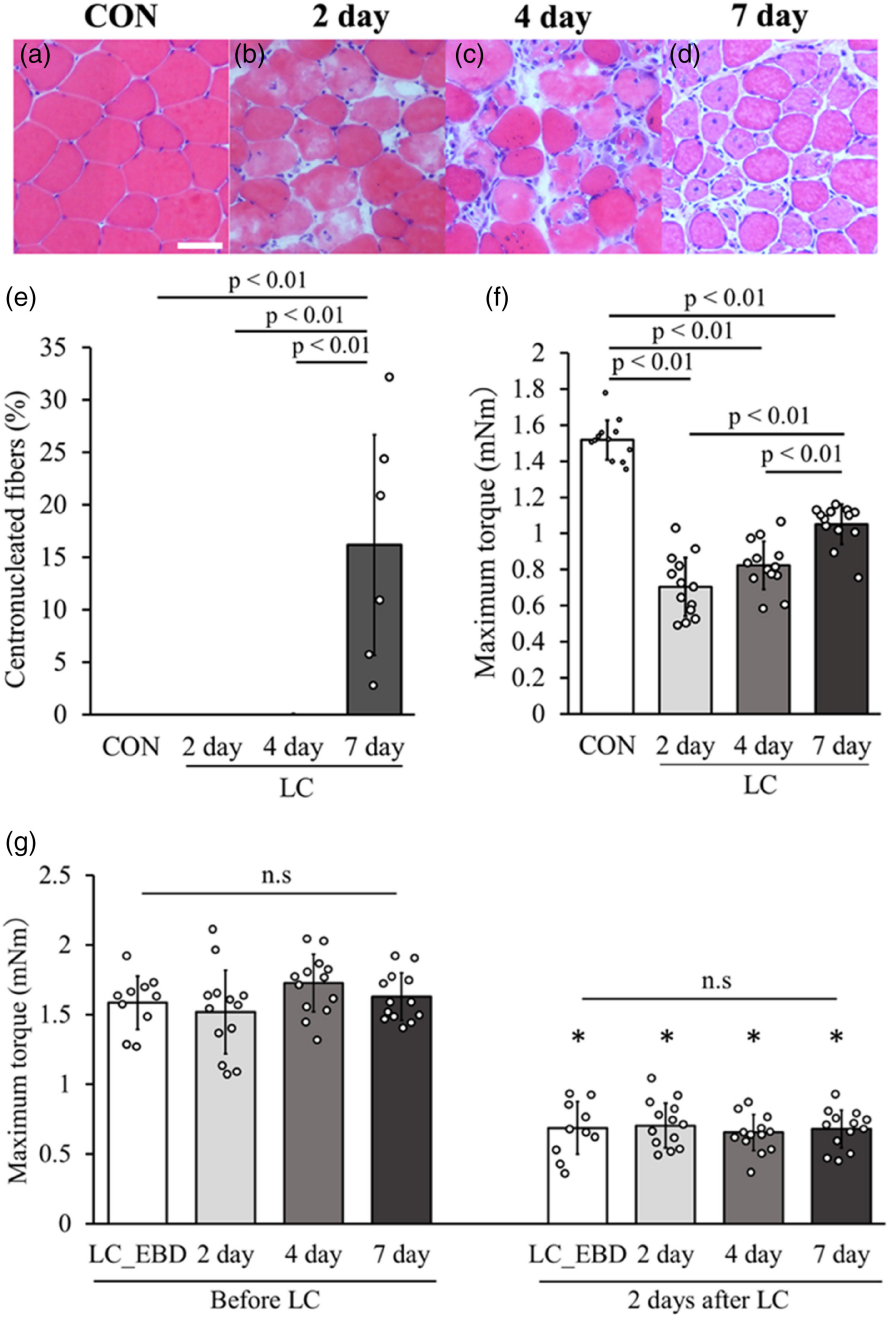
Changes in the skeletal muscle morphology and maximum torque after LC. H–E‐stained image after LC, with arrows indicating central nuclear fibers (a–d). Percentage of central nuclear fibers in the whole muscle belly (e), and maximum torque for each group (f). Maximum torque before LC and 2 days after LC for the LC_EBD, 2‐day, 4‐day, and 7‐day groups (g). Scale bars = 50 μm. *<0.05; statistical differences from before LC in the same group. Data are presented as means ± SD.

The 2‐day group showed lighter‐colored myofibers than the surrounding fibers and nuclear influx into the myofibers in the H–E staining images (Figure 3b). In the 4‐day group, there was an accumulation of nuclei in the area of the damaged myofibers (Figure 3c). In the 7‐day group, central nuclear fibers, which are smaller in size than normal myofibers and have nuclei in the central part of the myofibers, were observed (Figure 3d) in 16.2% ± 10.5%, but seldomly observed in the other groups (Figure 3e). The maximum torque of the LC group was significantly lower than that of the CON group (1.52 ± 0.11 mNm) (Figure 3f). However, the maximum torque value in the 7‐day group (1.05 ± 0.11 mNm) was significantly higher than that in the 2‐day (0.70 ± 0.16 mNm) and 4‐day (0.82 ± 0.13 mNm) groups. The maximum torque values before LC in the 2‐day (1.52 ± 0.30 mNm), 4‐day (1.73 ± 0.21 mNm), and 7‐day (1.63 ± 0.17 mNm) groups did not differ significantly compared with those before LC in the LC_EBD group. Furthermore, the maximum torque values after LC in the 2‐day (0.70 ± 0.16 mNm), 4‐day (0.65 ± 0.13 mNm), and 7‐day (0.68 ± 0.14 mNm) groups did not differ significantly compared with those in the LC_EBD group 2 days after LC, and all groups showed significantly lower values than the maximum torque before LC (Figure 3g).

### 3.3 Histological changes in lymphatic vessels and blood capillaries

**FIGURE 4:**
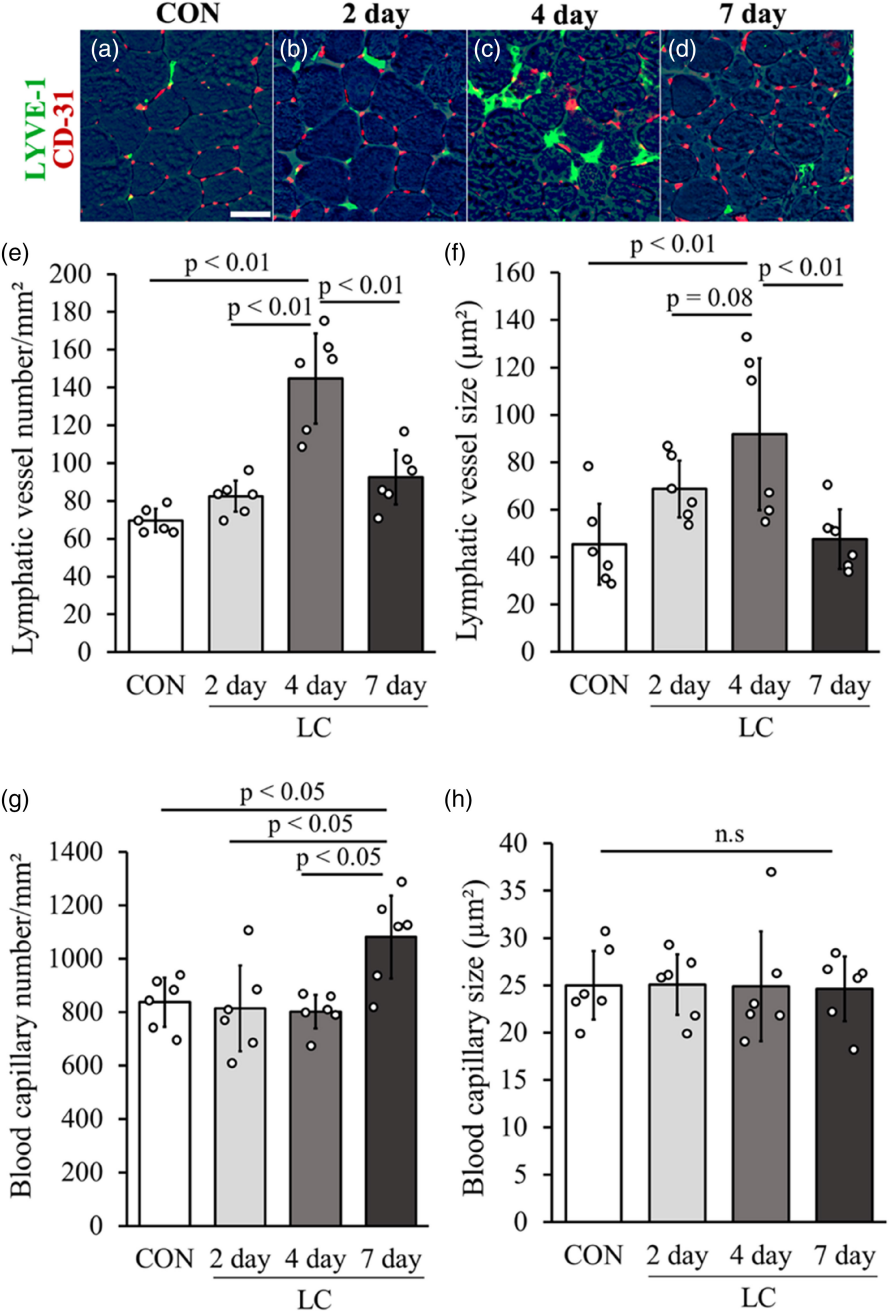
Histological changes in lymphatic vessels and blood capillaries after LC. Immunohistochemically stained images (green: LYVE‐1; red: CD‐31) after LC, superimposed on phase‐contrast photographed myofibers (a–d). Graphs present the number of lymphatic vessels per unit area (e), average area of lymphatic vessels (f), number of blood capillaries per unit area (g), and average area of blood capillaries (h). Scale bars = 50 μm. Data are presented as means ± SD.

The number of lymphatic vessels in the 4‐day group (144.8 ± 23.9) was significantly higher than that in the other groups (Figure 4e). There was no significant difference in the number of lymphatic vessels in the CON group (69.9 ± 6.2), 2‐day group (82.4 ± 8.2), and 7‐day group (92.6 ± 14.5). The mean area of lymphatic vessels in the 4‐day group (91.8 ± 32.1 μm^2^) was significantly higher than that in the CON group (45.3 ± 17.1 μm^2^) and 7‐day group (47.5 ± 12.6 μm^2^), and tended to be larger than that in the 2‐day group (68.7 ± 11.9 μm^2^) (*p* = 0.08, Figure 4f). There was no significant difference in the value of the mean area of lymphatic vessels in the CON, 2‐day, and 7‐day groups. The number of blood capillaries in the 7‐day group (1081.1 ± 155.2) was significantly higher than that in the other groups. There was no significant difference in the number of blood capillaries among CON (836.9 ± 91.8), 2‐day (813.8 ± 160.8), and 4‐day groups (800.9 ± 63.1) (Figure 4g). The mean area of blood capillaries was not significantly different between each group (CON group: 25.0 ± 3.6 μm^2^, 2‐day group: 25.1 ± 3.2 μm^2^, 4‐day group: 24.9 ± 5.8 μm^2^, 7‐day group: 24.6 ± 3.4 μm^2^) (Figure 4h). Furthermore, no significant differences were observed in the total muscle cross‐sectional area of the TA muscle among the groups (CON group: 7.3 ± 0.7 mm^2^; 2‐day group: 7.3 ± 0.6 mm^2^; 4‐day group: 6.4 ± 0.7 mm^2^; and 7‐day group: 6.5 ± 0.6 mm^2^; Figure S1).

### 3.4 Histological changes in macrophages

**FIGURE 5:**
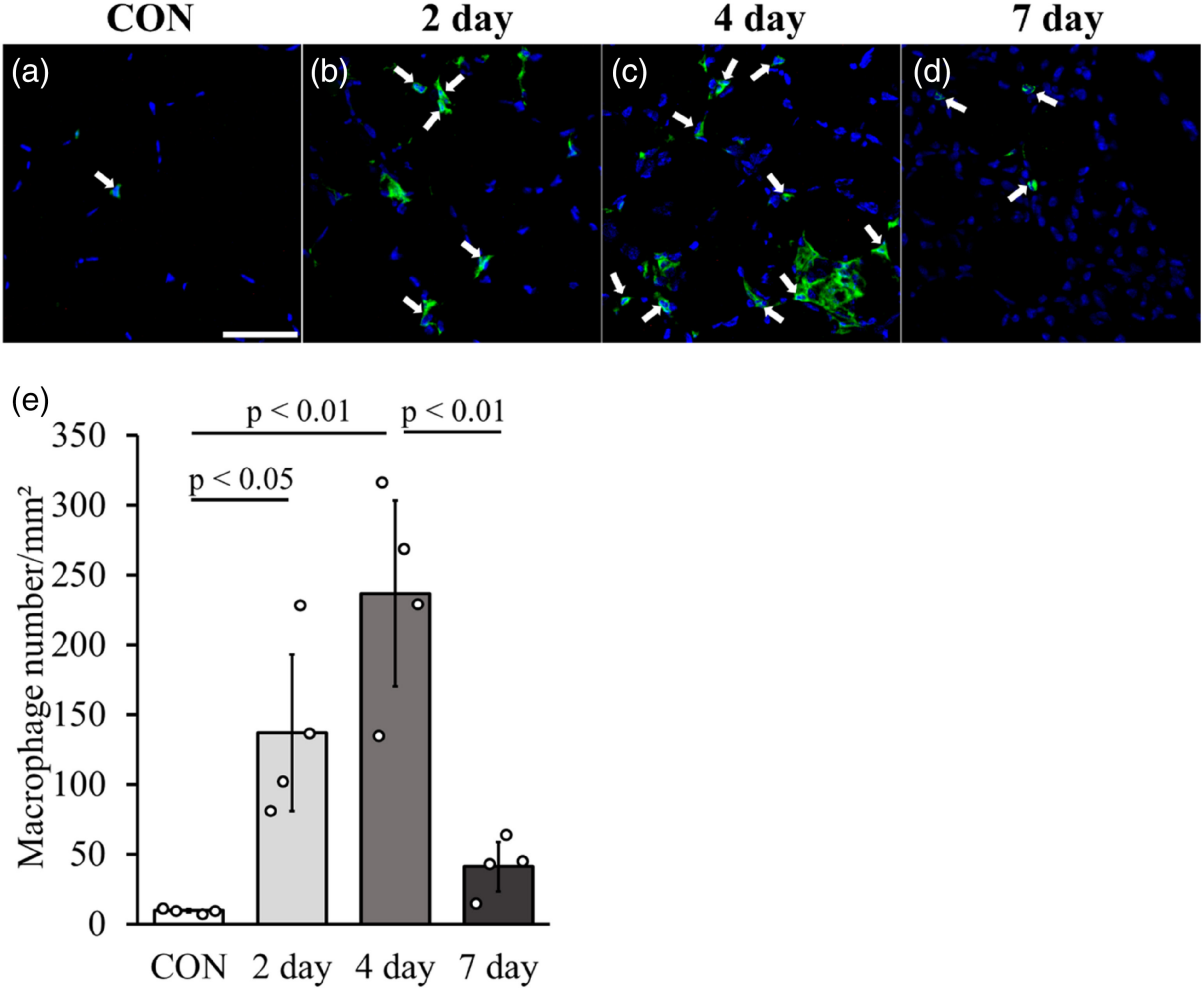
Histological changes in macrophages after LC. Immunohistochemically stained images (green: F4/80, blue; DAPI) after LC. As indicated by the arrow, F4/80‐positive cells (green) surrounding or adjacent to the nucleus (blue) were defined as macrophages (a‐d). The graph shows the number of macrophages per unit area (e). Scale bars = 50 μm. Data are presented as means ± SD.

The number of macrophages in the 4‐day group (236.9 ± 66.5) was significantly higher than that in both CON (9.8 ± 1.2) and 7‐day (41.2 ± 17.7) groups. However, there was no significant difference in the number of macrophages between the 2‐day group (137.0 ± 56.2) and the 4‐day group (Figure 5e). Conversely, the number of macrophages in the 2‐day group was significantly higher than that in the CON group. Notably, there was no significant difference in the number of macrophages between the CON and 7‐day groups.

### 3.5 Changes in mRNA levels of vascular endothelial growth factor

**FIGURE 6:**
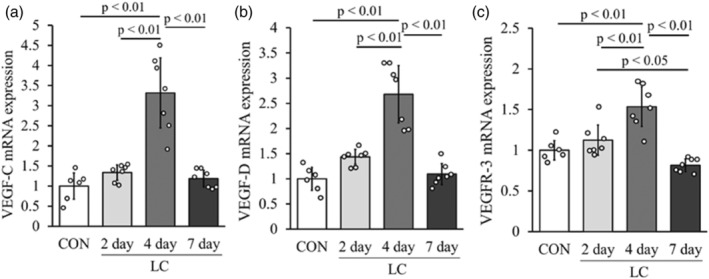
Changes in messenger RNA expression of VEGF‐C, VEGF‐D, and VEGFR‐3 after LC. Changes in mRNA expression of VEGF‐C (a), VEGF‐D (b), and VEGFR‐3 (c) in each group (normalized by GAPDH). Values represent fold change relative to the CON group. Data are presented as means ± SD.

The mRNA levels of VEGF‐C, VEGF‐D, and VEGFR‐3 in the 4‐day group were significantly higher than those in the other groups (Figure 6). There were no significant differences in the mRNA levels of VEGF‐C and VEGF‐D in the CON, 2‐day, and 7‐day groups (Figure 6a,b). In contrast, the mRNA level of VEGFR‐3 in the 2‐day group was significantly higher than that in the 7‐day group. There was no significant difference in VEGFR‐3 mRNA levels between the CON group and the 7‐day group (Figure 6c).

### 3.6 Changes in mRNA levels of pro‐inflammatory cytokines

**FIGURE 7:**
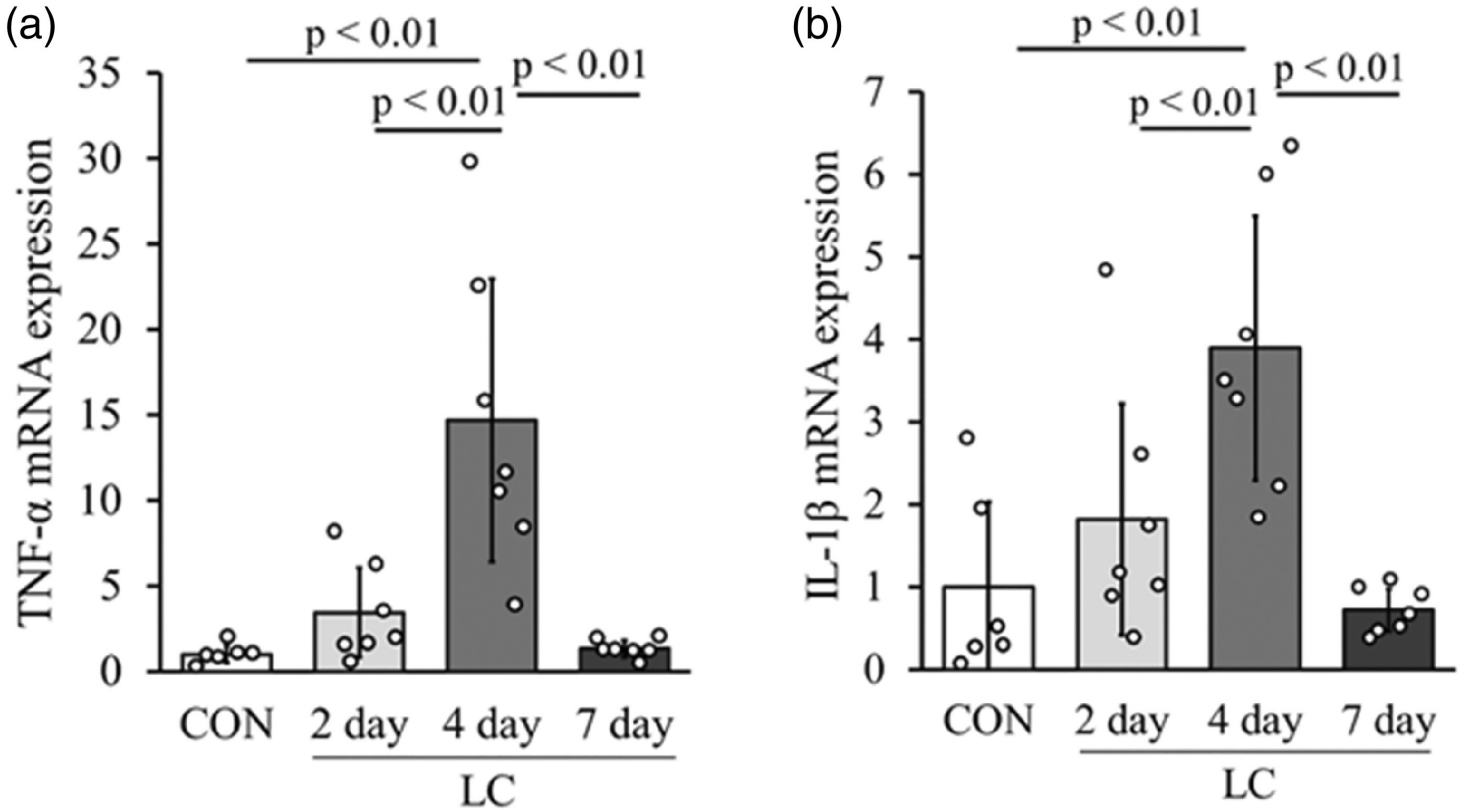
Changes in messenger RNA expression of TNF‐α and IL‐1β after LC. Changes in mRNA expression of TNF‐α (a) and IL‐1β (b) in each group (normalized by GAPDH). Values represent fold changes relative to the CON group. Data are presented as means ± SD.

The mRNA expression levels of TNF‐α and IL‐1β in the 4‐day group were significantly higher than those in the other groups (Figure 7). There were no significant differences in the mRNA expression levels of TNF‐α and IL‐1β in the CON, 2‐day, and 7‐day groups (Figure 7a,b).

## 4 DISCUSSION

In this study, we investigated the morphological and functional changes of muscular tissues and lymphatic vessels by using our established mouse model of LC‐induced skeletal muscle injury. However, the extent to which the LC induces skeletal muscle damage remains uncertain. Therefore, in this model, we estimated the extent of skeletal muscle damage through quantitative assessment via EBD labeling to measure damaged muscle fibers and conduct functional assessment based on maximum torque values. It has been reported that EBD binds to serum albumin and flows into myofibers with increased membrane permeability, such as myofibers in *mdx* mice (a mouse model of muscular dystrophy) and those with damaged cell membranes due to mechanical stimulation (Hamer et al., 2002; Matsuda et al., 1995; Mori et al., 2014; Tidball et al., 1999). In the present study, EBD‐positive myofiber at 2 days after injury are characterized by decreased staining in H–E staining and accumulation of macrophages. Based on the abovementioned findings, we defined EBD‐positive myofibers as damaged myofibers (Figure 2a). As there were no significant differences in maximum torque values before and 2 days after LC among LC_EBD, 2‐day, 4‐day, and 7‐day groups, it can be inferred that approximately 15% of the muscle fibers were injured by LC in the 2‐day, 4‐day, and 7‐day groups. Moreover, we anticipate that this model will prove highly beneficial for future research, especially in assessing the effectiveness of treatments for muscle damage in 10‐week‐old C57/BL6J male mice.

In H–E stained images, we observed damaged myofibers 2 and 4 days after LC, accompanied with the simultaneous accumulation of cells within the damaged myofibers. Regarding cell accumulation following eccentric contraction‐induced muscle injury, Kawashima et al. reported macrophage accumulation and minor proliferation of muscle satellite cells in the injured region of the gastrocnemius muscle in mice subjected to eccentric contraction (Kawashima, Kawanishi, et al., 2021). Additionally, Abigail et al. reported macrophage activation in the injured muscle following electrical stimulation‐induced eccentric contraction (Mackey & Kjaer, 2017). In our muscle injury model, there was a significant accumulation of macrophages 2 and 4 days after LC. Therefore, we hypothesized that most cells accumulating in the muscle injury region in our model are inflammatory cells, particularly macrophages. However, at 7 days after LC, the central nucleus myofibers involved in regeneration were observed. The maximum torque as a functional evaluation was significantly higher 7 days after LC than 2 or 4 days after LC. These results suggest that the muscles at 7 days after LC showed a trend toward recovery both histologically and functionally.

Previous research on the distribution and structure of lymphatic vessels in skeletal muscle suggests that lymphatic vessel density either remains stable or decreases after exercise (Greiwe et al., 2016; Kivelä, Silvennoinen, et al., 2007). However, these studies have only focused on the effects of long‐term post‐exercise, which is a different phenomenon from the acute inflammation following skeletal muscle injury examined in this study. In other words, the present results that the number of intramuscular lymphatic vessels increases rapidly during acute inflammation following skeletal muscle injury are new and crucial findings. In particular, during inflammation, marked alterations in the distribution and morphology of lymphatic vessels in the tissues are evident. The increase of lymphatic vessels during inflammatory processes has been reported in dermatitis (Huggenberger et al., 2010) and arthritis (Zhang et al., 2007). In a mouse model of dermatitis in ears and back skins treated with oxazolone, the number of lymphatic vessels in ears and back skins increased after 21 days of inflammation (Huggenberger et al., 2010), and in an arthritis model using TNF‐transgenic mice the number of lymphatic vessels in the synovium increased after 14 days of inflammation (Zhang et al., 2007). The present result suggests that the increase in lymphatic vessels during inflammation after skeletal muscle injury occurs in a shorter period than in dermatitis and arthritis. The reason for this difference may be that skeletal muscle is an organ that changes in size and shapes more easily than skin or joints. For example, skeletal muscle size is reduced by two‐thirds after 2 weeks of tail suspension (Eash et al., 2007; Kawashima, Ji, et al., 2021) and then returns to normal after 1 week of training (Itoh et al., 2017). To maintain the internal environment of the muscle, which is rich in such histological changes, we think that the lymphatic vessels within the muscle function in such a way that they can respond by increasing or decreasing their number.

To investigate the mechanism of the increase in lymphatic vessels in the skeletal muscle injury model, we examined the mRNA expression levels of VEGF‐C, VEGF‐D, and their receptor VEGFR‐3, which are lymphatic endothelial growth factor. These factors are upregulated in tumor tissues (Skobe et al., 2001; Stacker et al., 2001) and dermatitis (Huggenberger et al., 2010), and arthritis models (Guo et al., 2009; Zhang et al., 2007) are considered to be important factors inducing lymphangiogenesis. Previous studies have also reported that when muscle atrophy occurred by the tail suspension, VEGF‐C mRNA expression was reduced and the number of lymphatic vessels was also reduced (Kawashima, Ji, et al., 2021). Thus, as in dermatitis or arthritis, the expression of these factors was directly involved in the histological changes of the lymphatic vessels during skeletal muscle injury. In addition, inflammatory cytokines (TNF‐α, IL‐1β, TLR‐4, and NFκB) are factors involved in changes in the expression of lymphatic endothelial cell growth factors (Ji, 2012). TNF‐α and IL‐1β are released from inflammatory macrophages (M1 macrophages) that accumulate around damaged myofibers during skeletal muscle injury (Liu et al., 2017; Xiao, Liu, & Chen, 2016; Xiao, Liu, Luo, et al., 2016). In this study, based on the histological image on 4 days after LC, macrophage accumulation was observed in the damaged myofibers. These findings suggest that LC induces accumulation of macrophages in damaged myofibers and secretion of TNF‐α and IL‐1β and induces expression of VEGF‐C, VEGF‐D, and VEGFR‐3, increasing lymphatic vessels. In contrast, the histology on 7 days after LC showed almost no accumulation of macrophages, and the expression levels of TNF‐α and IL‐1β had returned to CON group levels.

Moreover, the expression levels of TNF‐α and IL‐1β decreased, and the number of lymphatic vessels returned to their original state. In other words, the number of lymphatic vessels was also reduced as a result of decreased expression of inflammatory cytokines that induce these lymphatic endothelial cell growth factors. However, further validation of these mechanisms by inhibition experiments to inhibit lymphangiogenesis after skeletal muscle injury is needed to confirm these mechanisms.

The present study revealed that the size of lymphatic vessels also increased significantly 4 days after LC. The result may suppose that the expansion of lymphatic vessels may have resulted from increased expression of lymphatic endothelial cells due to inflammation, similar to the mechanism of lymphatic vessel increase (Huggenberger et al., 2010; Zhang et al., 2007). However, some reports suggest that the lymphatic endothelial growth factor is not involved in lymphatic vessel expansion (Huggenberger et al., 2011). Where tissue fluid pressure may contribute to the dilation of lymphatic vessels (Planas‐Paz et al., 2012), and further research is needed to elucidate the mechanism by which lymphatic vessels expand.

As described above, it is considered that intramuscular lymphatic vessels after skeletal muscle injury undergo morphological changes such as increase and expansion earlier than the regeneration of myofibers, and return to their original state at the time when the central nuclear fibers grows and maximal torque improves. In addition, an increase in blood capillaries was observed during the period of myofiber regeneration. These changes are reasonable considering the timing of clearance of damaged tissues, and the transport of substances and energy necessary for metabolic activities during the recovery process.

Blood capillaries play a pivotal role in delivering oxygen and nutrients to the skeletal muscle, making them essential for recovery from muscle injury (Chongsatientam & Yimlamai, 2016; Pu et al., 2021). In general, VEGF‐A and its receptor VEGFR‐2 (Melincovici et al., 2018) are associated with angiogenesis, although some studies have suggested the involvement of VEGF‐D. Karkkainen et al. demonstrated that human VEGF‐D transgenic mice induce intramuscular angiogenesis but not lymphangiogenesis (Karkkainen et al., 2009). In contrast, it has been reported that mouse VEGF‐D specifically binds to VEGFR‐3, a factor associated with lymphangiogenesis, rather than VEGFR‐2, a factor linked to angiogenesis (Baldwin et al., 2001). In our study, compared with the control group, the mRNA expression level of VEGF‐D at 7 days after LC exhibited no significant difference, suggesting that VEGF‐D likely did not contribute to blood capillary growth.

Recent insights indicate that lymphangiogenesis affects angiogenesis inhibitory factors, including angiostatin, endostatin, and vasohibin (Pu et al., 2021). Moreover, the promotion of lymphangiogenesis after skeletal muscle injury may induce the promotion of angiogenesis and contribute to the differentiation of muscle stem cells. In recent years, symbiosis phenomena due to crosstalk between various tissues have been reported (Giudice & Taylor, 2017; Severinsen & Pedersen, 2020). We think that lymphangiogenesis in muscles contributes to various cell processes involved in muscle regeneration, such as inducing the proliferation and differentiation of muscle satellite cells, which may affect functional recovery from skeletal muscle injury. Indeed, it is necessary to clarify these causal relationships at times besides the 2, 4, and 7 days after LC and the detailed information of the inhibition experiments of lymphangiogenesis is especially needed in this new study field.

In conclusion, we created a mouse model of LC‐induced skeletal muscle injury that simulates the trauma caused by excessive strain in daily life and sports, and clarified the morphological changes of intramuscular lymphatic vessels and blood capillaries during the recovery process from the injury. We observed macrophage accumulation, increase in the levels of inflammatory cytokines (TNF‐α and IL‐1β), increased expression of lymphatic endothelial growth factors, and increased number and expansion of intramuscular lymphatic vessels 2–4 days after LC. In addition, regeneration of myofibers and expansion of intramuscular blood capillaries were observed 7 days after LC. Although more detailed verification of the timing and causal relationship is needed, we think that lymphangiogenesis plays an important role in recovery from skeletal muscle injury. The present findings are important for the development of new physiotherapy for skeletal muscle injury.

## FUNDING INFORMATION

This work was supported by the Japan Society for the Promotion of Science under the grant numbers KAKENHI JP22H03440 (to K.K.), JP17K01511 (to R.J.) JP20K11156 (to R.J.), JP21K19722 (to K.K.), JP19K11398 (to Y.I.), and JP22K11351 (to Y.I.).

## CONFLICT OF INTEREST STATEMENT

We have no conflict of interest with regard to our research.

## ETHICS STATEMENT

We confirm that we have read the Journal's position on issues involved in ethical publication and affirm that this report is consistent with those guidelines.

## Supporting information


Table S1.

Figure S1.
