# Supplementary material for: Histological and biochemical changes in lymphatic vessels after skeletal muscle injury induced by lengthening contraction in male mice
Source: Physiol Rep. 2024 Feb 14;12(3):e15950. doi: 10.14814/phy2.15950 (PMC10866689; doi:10.14814/phy2.15950)
Supplement: Supplementary file 1 — Table S1. [file PHY2-12-e15950-s001.docx]

| **Supplemental Table S1 Primers for real-time RT–PCR** | |
| --- | --- |
| **Gene** | **SYBR Green Primer (5′-3′)** |
| **VEGF-C** | Fwd: GTAAAAACAAACTTTTCCCTAATTC |
|  | Rev: TTTAAGGAAGCACTTCTGTGTGT |
| **VEGF-D** | Fwd: GCAAGACGAGACTCCACTGC |
|  | Rev: GGTGCTGAATGAGATCTCCC |
| **VEGFR-3** | Fwd: GCAGGAGGAGGAAGAGGAGC |
|  | Rev: TGCATGCTGGGTGGACTATCA |
| **TNF-α** | Fwd: TCTCATTCCTGCTTGTGGC |
|  | Rev: CACTTGGTGGTTTGCTACG |
| **IL-1β** | Fwd: TCCAGGATGAGGACATGAGCAC |
|  | Rev: GAACGTCACACACCAGCAGGTTA |
| **GAPDH** | Fwd: GACGGCCGCATCTTCTTGTG |
|  | Rev: CTTCCCATTCTCGGCCTTGACTGT |
