# Supplementary material for: Histological and biochemical changes in lymphatic vessels after skeletal muscle injury induced by lengthening contraction in male mice
Source: Physiol Rep. 2024 Feb 14;12(3):e15950. doi: 10.14814/phy2.15950 (PMC10866689; doi:10.14814/phy2.15950)
Supplement: Supplementary file 2 — Figure S1. [file PHY2-12-e15950-s002.docx]

**
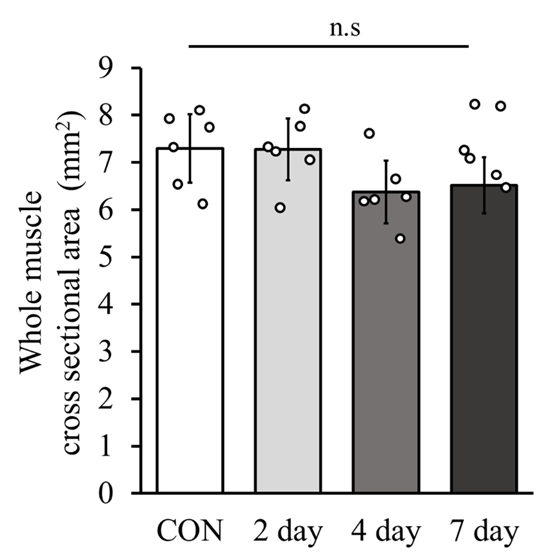
**

**Supplemental Fig. S1 Whole muscle cross sectional area**

The whole cross-sectional area of the TA muscle is shown (mean ± SD).
